# Patient Self-Assessment of Walking Ability and Fracture Risk in Older Australian Adults

**DOI:** 10.1001/jamanetworkopen.2023.52675

**Published:** 2024-01-23

**Authors:** Dana Bliuc, Thach Tran, Dunia Alarkawi, Weiwen Chen, Dima A. Alajlouni, Fiona Blyth, Lyn March, Robert D. Blank, Jacqueline R. Center

**Affiliations:** 1 Skeletal Diseases Program, Garvan Institute of Medical Research, Sydney, New South Wales, Australia; 2 School of Population Health, Faculty of Medicine and Health, UNSW, Sydney, New South Wales, Australia; 3 UNSW Ageing Future Institute, Sydney, New South Wales, Australia; 4 St Vincent’s Clinical School, Faculty of Medicine and Health, UNSW, Sydney, New South Wales, Australia; 5 Concord Clinical School, University of Sydney, Sydney, New South Wales, Australia; 6 Institute of Bone & Joint Research, University of Sydney, Sydney, New South Wales, Australia

## Abstract

**Importance:**

The relationship between self-reported walking limitation, a proxy of muscle function, and fracture risk has not been investigated.

**Objective:**

To examine the association between a self-reported walking limitation of 1000 m or less and 5-year risk of fracture.

**Design, Setting, and Participants:**

This prospective cohort study compared individuals with various degrees of walking ability limitation at 1000 m (a little limitation and a lot of limitation) and those without limitation (no limitation) accounting for age, falls, prior fractures, and weight. Participants from the ongoing population-based Sax Institute 45 and Up Study were followed from recruitment (2005-2008) for 5 years (2010-2013). Data analysis was conducted from July 2020 to September 2023.

**Exposure:**

Self-reported walking limitation.

**Main Outcomes and Measures:**

Incident fracture and site-specific fractures (hip, vertebral, and nonhip nonvertebral [NHNV] fractures).

**Results:**

Among the 266 912 participants enrolled in the 45 and Up Study, 238 969 were included, with 126 015 (53%) women (mean [SD] age, 63 [11] years) and 112 954 (47%) men (mean [SD] age, 61 [11] years). Approximately 20% reported a degree of limitation in walking 1000 m or less at baseline (39 324 women [24%]; 23 191 men [21%]). During a mean (SD) follow-up of 4.1 (0.8) years, 7190 women and 4267 men experienced an incident fracture. Compared with participants who reported no walking limitations, a little limitation and a lot of limitation were associated with higher risk of fracture (a little limitation among women: hazard ratio [HR], 1.32; 95% CI, 1.23-1.41; a little limitation among men: HR, 1.46; 95% CI, 1.34-1.60; a lot of limitation among women: HR, 1.60; 95% CI, 1.49-1.71; a lot of limitation among men: HR, 2.03; 95% CI, 1.86-2.22). Approximately 60% of fractures were attributable to walking limitation. The association was significant for hip, vertebral, and NHNV fracture and ranged between a 21% increase to a greater than 219% increase.

**Conclusions and Relevance:**

In this cohort study of 238 969 participants, self-reported walking limitations were associated with increased risk of fracture. These findings suggest that walking ability should be sought by clinicians to identify high-risk candidates for further assessment.

## Key Points

**Question:**

Are adults aged 45 years and older with a walking ability limitation at a higher fracture risk compared with same-age adults without a walking limitation?

**Findings:**

In this cohort study with 238 969 persons, 1 in 5 reported a limitation in walking 1000 m or less. Walking limitation was significantly associated with between a 32% and 219% higher fracture risk and contributed to approximately 60% of fractures.

**Meaning:**

In this study, self-reported walking limitations were common; given that they are easily detected, they should be sought by clinicians to identify high-risk candidates for further bone assessment.

## Teaser

This cohort study examines the association between a self-reported walking limitation and 5-year risk of fracture among Australian adults older than 45 years.

## Introduction

Fractures affect approximately 40% of women and 25% of men older than 60 years,^1^ and their incidence will increase exponentially over the next decades.^2^ The personal burden of fractures encompasses an increased risk of disability, loss of independence,^3^ refracture,^4^ and premature mortality.^5,6,7,8^

Individuals are currently identified as being at high risk of fracture through the use of risk calculators (such as Garvan and the Fracture Risk Assessment Tool [FRAX]) that include clinical risk factors and bone mineral density (BMD).^9,10,11^ Although these calculators have been validated and have good predictive ability, they are suboptimal.^12,13,14^

Muscle function is tied to fracture risk not only due to its association with the risk of falls but also due to its intimate association with bone loss, which is one of the strongest fracture risk predictors.^15,16^ It is well recognized that bone and muscle tissues communicate via paracrine and endocrine signals to coordinate the response to loading and injury across the whole lifespan. In the aging process, bone loss and muscle loss tend to occur simultaneously, leading to increased risk of falls, fractures, and mortality.^15,16^ Furthermore, several epidemiological studies have demonstrated that physical activity is associated with a reduction in the risk of fracture in both women and men.^17,18^

Given their contribution to fracture risk, muscle parameters are currently investigated for their role in fracture prediction tools.^19,20^ However, integration of measures of muscle function into the existent risk calculators has a number of limitations. The association between muscle strength and performance and fracture risk is weaker or not statistically significant in women.^16,21^ Clinical measures of muscle mass (primarily dual-energy x-ray absorptiometry–derived) are not accurate and have limited value in fracture risk prediction.^16^ Furthermore, individuals who are unable to perform muscle performance tests have the highest fracture risk.^19,22^

Walking ability is a predictor of poor outcomes including acute cardiovascular events and premature mortality.^23^ This study hypothesized that a simple qualitative measure of walking ability would be associated with fracture risk. The primary aim was to investigate the association between self-reported limitation in the ability to walk for 1000 m or less (equivalent of 1440 steps for an 162-cm tall woman and 1371 steps for a 176-cm tall men) and 5-year risk of fracture. The secondary aim quantified the association between self-reported walking limitation and 5-year risk of fracture at specific anatomical sites (hip, vertebral, and nonhip nonvertebral [NHNV] fractures).

## Methods

### Study Design

The study population consisted of 238 969 women and men participating in the Sax Institute 45 and Up Study, an ongoing prospective cohort study of healthy aging in New South Wales through linkage of questionnaires and administrative health databases. A detailed description of the study design and recruitment has been published previously.^24^ Participants older than 45 years were randomly selected from Medicare enrollment database and recruited in 2005 to 2009 after signing an informed consent form (eMethods 1 in Supplement 1). The Sax Institute’s 45 and Up Study was approved by University of New South Wales Human Research Ethics Committee. This study followed the Strengthening the Reporting of Observational Studies in Epidemiology (STROBE) reporting guideline.

### Data Sources

Demographic data (age, sex, height, weight, date of recruitment), lifestyle, and socioeconomic factors were collected using a questionnaire. All participants had their medical records linked to emergency presentations (Emergency Department Data Collection [EDDC]), hospital admissions (Admitted Patient Data Collection [APDC]), and deaths records (Registry of Births, Deaths and Marriages [RBDM]).

### Exposure

Walking ability was ascertained by 3 questions “Does your health limit you in walking (i) one kilometre; (ii) half kilometre; and (iii) 100 metres?” Each of these questions had 3 possible choices: “No—Not Limited at All,” “Yes—Limited A Little,” and “Yes—Limited a Lot.” Participants who reported discrepant information, such as those who answered a lot or a little for limited mobility at 100 m and not at all for limited mobility at 500 m or 1000 m were deemed invalid and were not included in the analyses (n = 928).

For the primary analysis, participants were classified according to walking limitation for the distance of 1 km or less in 3 groups: not at all, a little, and a lot. For an easier interpretation, we have also converted this distance to the equivalent number of steps using the kilometers-to-steps calculator available online.^25^ We estimated that the distance of 1 km correspond to 1440 steps for a 162-cm tall woman and 1371 steps for a 176-cm tall man. For sensitivity analyses we created 4 mutually exclusive groups: no limitation, limitations at 1000 m, limitations at 500 m, and limitations at 100 m, with a little and a lot levels collapsed into 1 category.

### Outcome

#### Fracture Identification

Fractures were identified using *International Statistical Classification of Diseases and Related Health Problems, Tenth Revision *(*ICD-10*) and Systematised Nomenclature of Medicine-Clinical Terms (SNOMED-CT) codes from the APDC and EDDC data sets in conjunction with the Australian Classification of Health Interventions (ACHI) procedure codes as previously reported.^6^ The combination of these sources result in a greater than 90% accuracy of fracture identification.^26^ High trauma fracture was identified using *ICD-10* codes related to car crashes and falls from heights greater than standing height and excluded from analysis. Multiple fractures (ie >4 bones broken in 1 event) were also classified as high-trauma and excluded. Fingers, toes, and skull fractures were excluded.

Fractures were classified according to the recruitment date as prior fractures or incident fractures. The index fracture represented the first fracture during the first 5 years after recruitment. The index fracture was classified in 3 categories: hip, clinical vertebral, and NHNV. If more than 1 fracture occurred in the same event, fracture site was classified according to the most proximal site.

### Covariates

Weight, age, sex, comorbidities, medication, and falls were determined from the baseline questionnaire. Weight was assessed using the question*: *“About how much do you weigh?” Falls were collected using the question*: *“During the last 12 month how many times have you fallen to floor or ground?” We further classified falls into a binary variable: fallers (those who reported any falls in the last year) and nonfallers (those who did not report any falls). Prior fracture was defined as any low-trauma fracture recorded within 5 years prior to recruitment.

### Cohort Selection and Follow-Up

All participants with valid data for exposure were eligible. Given that the outcome measurement was assessed using linked administrative databases, there were no missing values for the main outcome measurement. However, weight and falls were missing in approximately 9% of participants. We have used multiple imputation by chained equations^27^ to impute the missing weight and falls data.

### Statistical Analysis

Given the significant differences in fracture risk between women and men, all analyses were stratified by sex.^28^ Statistical significance was defined as a 2-tailed *P* < .05.

#### Five-Year Risk of Any Fracture

The association between walking limitation of 1000 m or less and 5-year fracture risk was assessed using Cox proportional hazards models. The follow-up time was calculated from baseline until index fracture, death, or 5 years following enrollment, whichever came first. The proportionality hazards assumption was checked using Schoenfeld residuals.^29^ Model 1 was adjusted for Garvan fracture variables^30^ (ie, age, weight, prior fracture and falls) and model 2 for age, comorbidities, medication use, bisphosphonate use, private health insurance, smoking, nursing home residency, and education. Given that the prevalence of walking limitation is greater in older people, we performed further analysis stratified by age (<70 and ≥70 years). To account for competing risk of mortality, we performed a Fine-Gray subdistribution hazards model.^31^

Heuristic population attributable fraction was used to determine the contribution of mobility in conjunction with Garvan fracture variables: age (≥70 years), weight (≤50 kg for women and ≤70 kg for men), falls, prior fracture, and walking limitation of 1000 m or less. For these analyses, the response levels of a lot and a little were collapsed into one category.^32^ Population attributable risk was similar for women and men and is reported together. Similar Cox models were constructed for hip, vertebral, and NHNV fracture.

#### Sensitivity Analyses

We performed several sensitivity analyses: (1) a fracture risk model weighted for the inverse probability of exposure effects, (2) a fracture risk model stratified by different distances of walking limitations (1000 m, 500 m, and 100 m), and (3) an analysis that estimated the E-value to quantify the impact of unmeasured confounders on the study findings^33^ (eMethods 2 in Supplement 1). All analyses were performed in SAS version 9.4 (SAS Institute).

## Results

**Figure 1.**
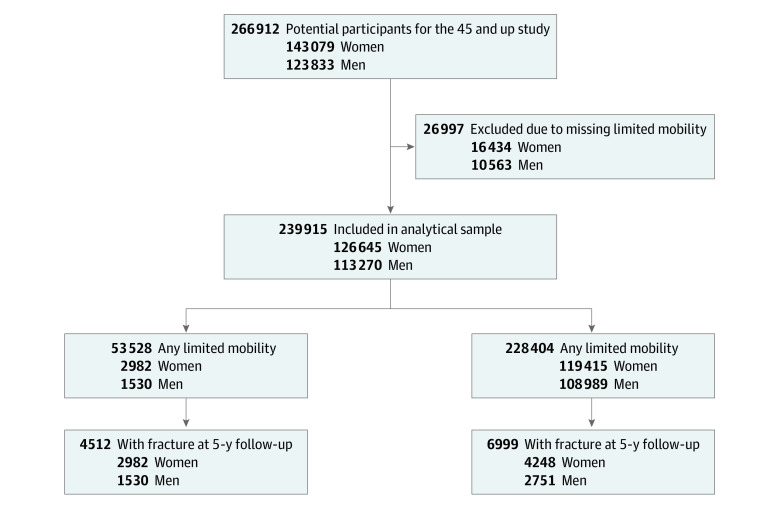
Flowchart of Study Participants

Of all 266 912 participants enrolled in the 45 and Up Study, 238 969 formed the analytical cohort, after excluding 27 943 participants due to missing or inconsistent values for walking mobility (Figure 1). Among these, 126 015 (53%) were women (mean [SD] age, 63 [11] years) and 112 954 (47%) were men (mean [SD] age, 61 [11] years). Approximately 24% of women (39 324) and 21% of men (23 191) reported a degree (either a lot or a little) of walking limitation for 1000 m or less. Those with walking limitations were older; had a higher prevalence of chronic conditions and medication use; and were more likely to smoke, live without a partner, and have a university education (Table 1).

**Table 1.** Baseline Characteristics According to Sex and Walking Limitation of 1000 m

Characteristic	Women (n = 126 015 [54%])				Men (n = 10 988 [46%])			
	Walking limitation, No. (%)			*P* value	Walking limitation, No. (%)			*P* value
	None (n = 95 691)	A little (n = 15 895)	A lot (n = 14 429)		None (n = 89 763)	A little (n = 12 838)	A lot (n = 10 353)	
Age, mean (SD), y	59 (9)	66 (12)	71 (13)	<.001	62 (10)	68 (11)	72 (12)	<.001
Weight, mean (SD), kg	69 (14)	75 (18)	75 (20)	<.001	84 (15)	87 (18)	87 (20)	<.001
Falls	15 055 (15.7)	4690 (29.5)	5845 (40.5)	<.001	8493 (9.5)	3061 (23.8)	3931 (38.0)	<.001
Prior fracture	10 185 (11.0)	2611 (16.4)	3108 (21.5)	<.001	6868 (7.7)	1314 (10.2)	1388 (13.4)	<.001
Baseline comorbidities								
Heart failure	5031 (5.0)	2314 (14.6)	3108 (21.5)	<.001	11 584 (12.9)	3481 (27.1)	3416 (33.0)	<.001
Diabetes	4599 (5.0)	1949 (12.3)	2584 (17.9)	<.001	7675 (8.6)	2268 (17.7)	2452 (23.7)	<.001
Parkinson disease	263 (0.3)	142 (0.9)	228 (1.6)	<.001	398 (0.4)	188 (1.5)	277 (2.7)	<.001
Anxiety	8979 (9.4)	2239 (14.1)	2098 (14.5)	<.001	4939 (5.5)	1184 (9.2)	1021 (9.9)	<.001
Asthma	9933 (10.0)	2686 (16.9)	2648 (18.4)	<.001	7004 (7.8)	1358 (10.6)	1166 (11.3)	<.001
Cancer	8208 (8.6)	1829 (11.5)	1812 (12.6)	<.001	5716 (6.4)	1107 (8.6)	918 (8.9)	<.001
Depression	13 615 (14.2)	3417 (21.5)	3209 (22.2)	<.001	7885 (8.8)	1907 (14.9)	1745 (16.9)	<.001
Dementia	866 (0.9)	296 (1.9)	415 (2.9)	<.001	1060 (1.2)	311 (2.4)	273 (2.6)	<.001
Stroke	1302 (1.4)	703 (4.4)	1317 (9.1)	<.001	2015 (2.2)	949 (7.4)	1236 (11.9)	<.001
Medications, mean (SD), No.	0.9 (1.2)	1.8 (1.6)	2.3 (1.8)	<.001	1.0 (1.3)	1.8 (1.7)	2.2 (1.9)	<.001
Bisphosphonates	2964 (3.1)	1060 (6.7)	1363 (9.5)	<.001	877 (1.0)	333 (2.6)	428 (4.1)	<.001
Nursing home residency	1467 (2.0)	412 (2.6)	843 (5.8)	<.001	1673 (1.9)	339 (2.6)	495 (4.8)	<.001
Smoking	5994 (6.3)	1348 (8.5)	1165 (8.1)	<.001	5949 (6.6)	1347 (10.5)	1072 (10.4)	<.001
Education								
School certificate	8209 (8.6)	2789 (17.6)	3567 (24.7)	<.001	8209 (9.2)	2323 (18.1)	2610 (25.2)	<.001
High school certificate	61 626 (4.4)	10 663 (67.1)	9102 (63.1)		55 508 (61.8)	8678 (67.6)	6587 (63.6)	
University education	24 916 (25.8)	2149 (13.5)	1341 (9.3)		26 046 (29.0)	1837 (14.3)	1156 (11.2)	

### Five-Year Risk of Any Fracture

During a mean (SD) follow-up of 4.1 (0.8) years, 7190 women and 4267 men experienced an incident fracture, yielding rates of 11.92 (95% CI, 11.65-12.20) fractures per 1000 person-years for women and 7.93 (95% CI, 7.70-8.18) fractures per 1000 person-years for men (eTable 1 in Supplement 1). In women, fracture risk increased significantly with increasing degree of walking impairment. In the model adjusted by Garvan variables (model 1), compared with no limitation, the a little limitation group had approximately 32% higher fracture risk (hazard ratio [HR], 1.32; 95% CI, 1.23-1.41), whereas the a lot of limitation group had approximately 60% higher fracture risk (HR, 1.60; 95% CI, 1.49-1.71) (Table 2; eFigure 1 in Supplement 1). The association remained statistically significant after adjustment for multiple confounding factors (model 2 in Table 2) as well as for the competing risk of mortality (a little limitation: adjusted subdistribution HR, 1.30; 95% CI, 1.21-1.39; a lot of limitation: adjusted subdistribution HR, 1.46; 95% CI, 1.36-1.57).

**Table 2.** Associations Between 1000-m Walking Limitation and Fracture Risk According to Sex^a^

Outcome	Hazard ratio (95% CI)					
	Women			Men		
	Unadjusted	Model 1	Model 2	Unadjusted	Model 1	Model 2
**Any fracture**						
Walking limitation						
Not at all	1 [Reference]	1 [Reference]	1 [Reference]	1 [Reference]	1 [Reference]	1 [Reference]
A little	1.89 (1.77-2.01)	1.32 (1.23-1.41)	1.22 (1.14-1.30)	1.88 (1.73-2.04)	1.46 (1.34-1.60)	1.39 (1.27-1.52)
A lot	3.05 (2.88-3.22)	1.60 (1.49-1.71)	1.35 (1.26-1.46)	3.11 (2.87-3.36)	2.03 (1.86-2.22)	1.90 (1.72-2.09)
**Hip fracture**						
Walking limitation						
Not at all	1 [Reference]	1 [Reference]	1 [Reference]	1 [Reference]	1 [Reference]	1 [Reference]
A little	4.70 (4.00-5.53)	2.19 (1.84-2.60)	1.91 (1.60-2.27)	4.68 (3.89-5.63)	2.41 (1.98-2.92)	2.09 (1.71-2.55)
A lot	10.59 (9.22-12.17)	2.63 (2.23-3.12)	2.12 (1.78-2.53)	9.06 (7.66-10.72)	3.34 (2.76-4.03)	2.61 (2.12-3.21)
**Vertebral fracture**						
Walking limitation						
Not at all	1 [Reference]	1 [Reference]	1 [Reference]	1 [Reference]	1 [Reference]	1 [Reference]
A little	2.61 (2.01-3.39)	1.59 (1.20-2.11)	1.28 (1.00-1.69)	2.19 (1.66-2.90)	1.41 (1.05-1.90)	1.47 (1.09-3.18)
A lot	5.72 (4.61-7.10)	2.15 (1.65-2.81)	1.63 (1.23-2.15)	4.47 (3.50-5.70)	2.21 (1.68-2.91)	2.36 (1.75-3.18)
**Nonhip nonvertebral fracture**						
Walking limitation						
Not at all	1 [Reference]	1 [Reference]	1 [Reference]	1 [Reference]	1 [Reference]	1 [Reference]
A little	1.61 (1.50-1.73)	1.21 (1.15-1.28)	1.28 (1.00-1.69)	1.50 (1.36-1.66)	1.30 (1.17-1.45)	1.24 (1.12-1.39)
A lot	2.28 (2.13-2.44)	1.36 (1.28-1.44)	1.63 (1.23-2.15)	2.27 (2.06-2.50)	1.72 (1.54 − 1.91)	1.66 (1.47-1.87)

^a^ Models 1 and 2 are adjusted for age; model 1 is adjusted for variables included in the Garvan fracture risk calculator (age, weight, prior fracture, falls); model 2 is adjusted for age, all comorbidities at baseline, number of medications, bisphosphonate use, marital status, education, and nursing home residency.

In men, fracture risk was significantly associated with walking limitation for both a little (HR, 1.46; 95% CI, 1.34-1.60) and a lot (HR, 2.03; 95% CI, 1.86-2.22) after adjustment for Garvan variables (model 1 in Table 2; eFigure 1 in Supplement 1) and declined but remained statistically significant after multiple confounding adjustment (model 2 in Table 2) and after adjustment for the competing risk of mortality (a little limitation: adjusted subdistribution HR, 1.42; 95% CI, 1.30-1.55; a lot of limitation: adjusted subdistribution HR, 1.80; 95% CI, 1.64-1.97). A further stratification by age found a slightly higher but not statistically significant magnitude of association in the older (≥70 years) compared with younger (<70 years) age groups in both sexes (eTable 2 in Supplement 1).

### Heuristic Population Attributable Fracture Risk

**Figure 2.**
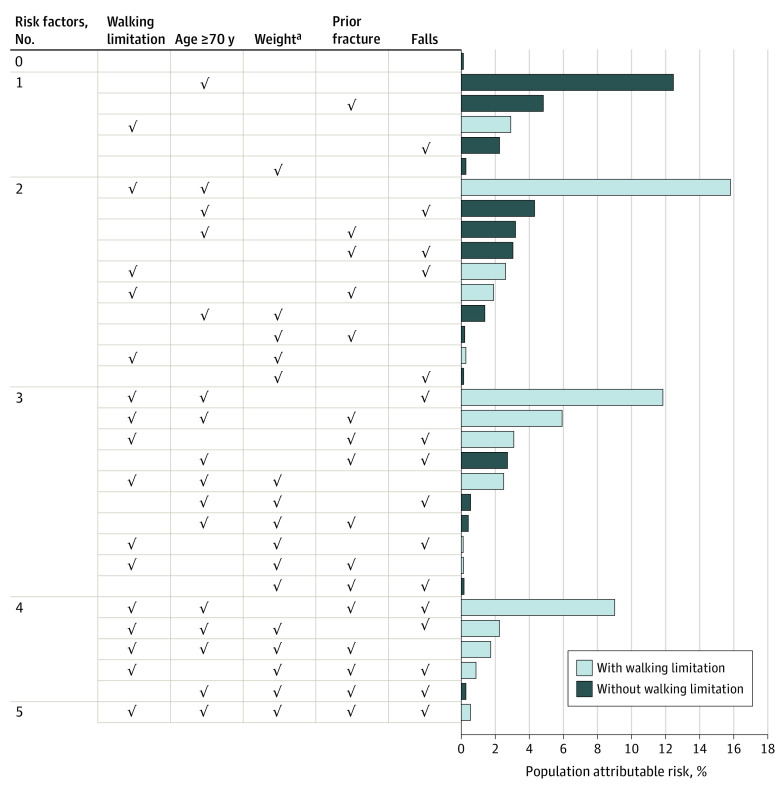
Heuristic Population Attributable Risk for Limited Mobility, Age, Weight, and History of Fracture and Falls ^a^Weight was defined as 50 kg or less for women and 70 kg or less for men.

Walking limitation was frequently associated with Garvan fracture variables. Given the high prevalence and magnitude of association with fracture risk, approximately 66% of fractures were attributable to walking limitation (Figure 2).

### Five-Year Risk of Specific Fracture Sites

**Figure 3.**
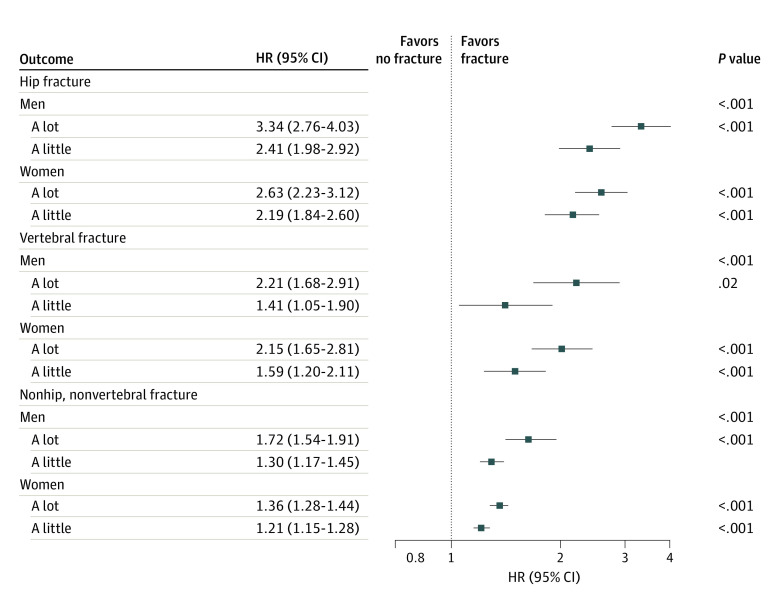
Forest Plot of Sex-Specific Hazard Ratios (HRs) Comparing the Risk of Different Fracture Sites According to Ability to Walk 1000 m

In women, compared with no limitation, those who reported a lot of limitation were associated with a greater than 2-fold higher risk of hip and vertebral fracture and a 36% higher risk of NHNV fractures and those with a little limitation had approximately 2-fold greater risk of hip and 21% to 59% greater risk of vertebral and NHNV fracture risk after adjustment for Garvan variables (model 1 in Table 2 and Figure 3). Similar findings, albeit with a weaker magnitude of association, were observed in the models adjusted for comorbidities, medication, education, and lifestyle factors (model 2 in Table 2).

In men, both degrees of walking limitation were also significantly associated with higher fracture risk at all sites. The group with a lot of limitation greater than 3-fold higher risk of hip fracture, greater than 2-fold higher risk of vertebral fracture, and 70% higher risk of NHNV fractures, and the group with a little had 2-fold higher risk of hip fracture and 28% to 39% higher risk of vertebral and NHNV fractures after adjustment for Garvan variables (model 1 in Table 2 and Figure 3). The magnitude of these associations declined, but remained statistically significant for all fracture outcomes after adjustment for comorbidities, medication, education, and lifestyle factors (model 2 in Table 2)

### Sensitivity Analyses

#### Five-Year Fracture Risk Weighted by Inverse Probability of Exposure

The association between 1000-m walking ability and fracture risk remained statistically significant in the Cox models weighted by the inverse probability of exposure. Specifically, women with a lot of and a little limitation walking 1000 m were associated with 19% (HR, 1.19; 95% CI, 1.11-1.28) and 16% (HR, 1.16; 95% CI, 1.09-1.24) increased fracture risk, respectively. In men, these associations were 1.64 (95% CI, 1.49-1.81) for a lot of limitation and 1.28 (95% CI, 1.16-1.40) for a little limitation in 1000-m walking.

#### Five-Year Fracture Risk for 1000-m, 500-m, and 100-m Walking Limitations

Of the 125 744 women, 14 494 (12%) reported limitations at 1000 m, 8456 (7%) at 500 m, and 7150 (6%) at 100 m. In men, of the total 117 247, 10 614 (9%) reported limitations at 1000 m, 6352 (6%) at 500 m, and 5803 (5%) at 100 m. Compared with individuals with no limitation, the prevalence of fracture risk factors (older age, lower weight, falls and prior fracture) as well as comorbidities increased with decreasing distance of walking limitation (eTable 3 in Supplement 1).

For both sexes, the magnitude of the association between fracture and different walking distances had a dose-response pattern (eTable 4 and eFigure 2 in Supplement 1). In women, fracture risk was increased by approximately 20% for 1000 m, approximately 43% for 500 m, and approximately 60% for 100 m. In men, a similar pattern was observed with fracture risk increased by approximately 32%, 62%, and more than 2-fold for 1000-, 500-, and 100-m distance limitations (eTable 4 and eFigure 2 in Supplement 1). The magnitude of these associations declined but maintained the dose-response pattern as well as statistical significance after multiple confounding adjustment (model 2 in eTable 4 in Supplement 1).

#### E-Value for 5-Year Fracture Risk

The E-value for the association between fracture and 1000-m walking limitation was 2.56 for a lot of limitation and 1.97 for a little limitation in women. Similar values for men were 3.46 for a lot of limitation and 2.28 for a little limitation.

This means that the residual confounding could explain the observed association if the unmeasured confounder has a relative risk of association with both fracture risk and walking limitation of at least 1.97 to 2.56 for women and 2.28 to 3.46 in men. While this may seem plausible, none of the strongest confounders in our study (ie, falls and prior fracture) reached such a magnitude of association with both the exposure and the outcome.

## Discussion

In this large population-based study of 238 969 women and men, self-reported limitation in the ability to walk was associated with increased risk of any fracture ranging between 32% and 219% independent of known fracture risk factors (eg, older age, lower weight, history of falls and prior fracture) in both sexes. The association between walking ability and fracture risk increased significantly between the degrees of limitation (a little limitation and a lot of limitation) and also between different distances of limitation (1000 m, 500 m, and 100 m), suggestive of a dose-response association.

Walking speed is integrated into complex geriatric constructs, such as frailty or sarcopenia definitions, with the aim of identifying high-risk individuals. However, both conditions are heterogenous,^34,35,36,37^ difficult to diagnose clinically, and their role in fracture risk is not clear.^38,39^ The novel finding of this study was that a simple self-reported qualitative measure of walking was consistently associated with fracture risk after adjustment for multiple confounding factors. Furthermore, it displayed a dose-response pattern with significant increase in fracture risk with decrease in the walking distance limitation (1000 m: HRs, 1.20-1.32; 500 m: HRs, 1.43-1.62; 100 m: HRs, 1.60-2.00), indicative of a potential cause-effect relationship.

Walking limitation affected approximately 1 in 5 participants and was frequently associated with known fracture risk factors (eg, lower weight, falls, prior fracture). Given its high prevalence and strong magnitude of association with fracture, walking limitation contributed to more than 60% of all fractures, suggesting that this simple measurement can be used by clinicians to identify high risk individuals.

Sedentary behavior has been associated not only with reduced muscle strength and performance, but also with risk of fracture and falls.^18,40^ An inverse association between leisure physical activity and hip fracture risk was found for both women and men.^41^ Nevertheless, this is the first study to our knowledge that addressed the role of inability to walk in fracture risk. Importantly, walking impairment can be reversible when related to aging, physical inactivity, and other treatable conditions.^42,43^ Targeted physical exercises could improve walking impairment.^44,45^ Further studies are needed to determine whether walking improvement will reduce the risk of fracture and mortality in the high-risk groups.

### Strengths and Limitations

This study has several strengths. First, the large sample size allowed in-depth analyses of fracture risk at all bone sites and degrees of walking limitations. Second, the availability of linked data ensured no missing value for fracture outcomes and no loss of follow-up. Third, the availability of known fracture risk factors allowed for important confounding adjustment.

However, the study has some limitations. The participants in the 45 and Up Study are healthier than the general population.^6^ Not surprisingly, the fracture rates in this study were lower than those reported in other population-based studies.^8,46,47^ However, it is expected that this selection bias will only underestimate the prevalence and the strength of association between walking limitation and fracture risk, as we are not capturing the population with more severe illness, among whom the effect size would be highest. Walking ability was not objectively measured and therefore could have been subject to measurement error. To minimize measurement error, we excluded all invalid data (ie, those who reported the ability to walk 1000 m but inability to walk 500 or 100 m). Furthermore, given that this error likely affected fracture and no-fracture participants equally, this information bias would only lead to an underestimation of our findings. It is also possible that the perception of walking ability is impacted in people experiencing cognitive impairment, mood disorders, and mental illnesses. We did not have information on cognitive function and mood disorders. However, we were able to adjust for the presence of mental illness. Nevertheless, the findings from our study may not be applicable to such patients. Additionally, the causes of walking impairment were not available in the study, and any inference on mechanistic pathways between walking limitation and fracture risk are speculative and based solely on a plausible biological mechanism.

## Conclusion

In this study, self-reported walking limitations were associated with increased risk of fracture among women and men. Limited walking ability can be sought by clinicians to identify high-risk candidates for further assessment (ie, BMD testing). Future studies are needed to see whether this simple evaluation could improve the existing fracture risk tools and whether measures to improve walking ability would have a beneficial effect on fracture risk.

## Notes

Supplement 1. **eMethods 1.** The 45 and Up Study Design **eMethods 2.** Sensitivity Analyses **eTable 1.** Incidence of Fractures According to Age and Sex **eTable 2.** Associations Between 1000 m Walking Limitation and Fracture Risk According to Sex and Age **eTable 3.** Baseline Characteristics for Participants Stratified According to 1000, 500, and 100 m Walking Distance Limitation **eTable 4.** Associations Between 100 m, 500 m, and 1000 m Walking Limitation and Fracture Risk According to Sex **eFigure 1.** Forest Plot of Sex-Specific Hazard Ratios Comparing Fracture Risk for 1000 m Walking Limitation **eFigure 2.** Forest Plot of Sex-Specific Hazard Ratios Comparing Fracture Risk According to Walking Limitation at Different Distances of 1000 m, 500 m, and 100 m

Supplement 2.
Data Sharing Statement
