# Error in Figure

**DOI:** 10.1001/jamanetworkopen.2024.2957

**Published:** 2024-02-19

In the Original Investigation titled “Patient Self-Assessment of Walking Ability and Fracture Risk in Older Australian Adults,”^1^ published January 23, 2024, there was an error in Figure 1. The box on the right-hand side that included 228 404 participants should have read “No limited mobility.” The article has been corrected.^1^
