# Supplementary material for: Patient Self-Assessment of Walking Ability and Fracture Risk in Older Australian Adults
Source: JAMA Netw Open. 2024 Jan 23;7(1):e2352675. doi: 10.1001/jamanetworkopen.2023.52675 (PMC10807297; doi:10.1001/jamanetworkopen.2023.52675)
Supplement: Supplement 1. — eMethods 1. The 45 and Up Study Design eMethods 2. Sensitivity Analyses eTable 1. Incidence of Fractures According to Age and Sex eTable 2. Associations Between 1000 m Walking Limitation and Fracture Risk According to Sex and Age eTable 3. Baseline Characteristics for Participants Stratified According to 1000, 500, and 100 m Walking Distance Limitation eTable 4. Associations Between 100 m, 500 m, and 1000 m Walking Limitation and Fracture Risk According to Sex eFigure 1. Forest Plot of Sex-Specific Hazard Ratios Comparing Fracture Risk for 1000 m Walking Limitation eFigure 2. Forest Plot of Sex-Specific Hazard Ratios Comparing Fracture Risk According to Walking Limitation at Different Distances of 1000 m, 500 m, and 100 m [file jamanetwopen-e2352675-s001.pdf]

Supplemental Online Content

Bliuc D, Tran T, Alarkawi D, et al. Patient self-assessment of walking ability and fracture risk in older Australian adults. *JAMA Netw Open.* 2024;7(1):e2352675. doi:10.1001/jamanetworkopen.2023.52675

eMethods 1. The 45 and Up Study Design

eMethods 2. Sensitivity Analyses

eTable 1. Incidence of Fractures According to Age and Sex

eTable 2. Associations Between 1000 m Walking Limitation and Fracture Risk According to Sex and Age

eTable 3. Baseline Characteristics for Participants Stratified According to 1000, 500, and 100 m Walking Distance Limitation

eTable 4. Associations Between 100 m, 500 m, and1000 m Walking Limitation and Fracture Risk According to Sex

eFigure 1. Forest Plot of Sex-Specific Hazard Ratios Comparing Fracture Risk for 1000 m Walking Limitation

eFigure 2. Forest Plot of Sex-Specific Hazard Ratios Comparing Fracture Risk According to Walking Limitation at Different Distances of 1000 m, 500 m, and 100 m

This supplemental material has been provided by the authors to give readers additional information about their work.

© 2024 Bliuc D et al. *JAMA Network Open.*

eMethods 1. The 45 and Up Study Design

Participants were randomly sampled from the Services Australia (formerly the Australian Government Department of Human Services) Medicare enrolment database, which provides near complete coverage of the population. The target population comprised women and men over 45 years of age with oversampling of the rural population and individuals aged over 80, to account for death and loss of follow-up. Recruitment took place between 2005 and 2009 and the response rate was 18%. While the study is ongoing, the follow-up for the analyses in this study were restricted to the first 5 years of follow-up (2010/2013).

© 2024 Bliuc D et al. *JAMA Network Open.*

eMethods 2. Sensitivity Analyses

(i) Inverse probability weighting for exposure effect

The first sensitivity analysis was performed to account for unequal distribution of baseline characteristics between exposed and unexposed[15]. The probability of being exposed (i.e., having a degree of walking limitation) was calculated for each individual participant using a logistic regression model that included age, weight, comorbidities, medication, falls, prior fracture, education, an index of remoteness and smoking. We have then generated weights for each individual as the inverse probability of receiving his/her actual exposure. Finally, a Cox's proportional hazards regression was conducted to examine the risk of fractures with applied inverse probability weights.

(ii) Fracture risk model stratified by different distances of walking limitations (1000m, 500m and 100m)

In order to determine whether there was any dose-effect between limited walking mobility and fracture risk, we conducted an age and multivariable Cox's proportional hazards model with participants classified in 4 mutually exclusive groups: no limitation (referent), and limitations at 1000 m, 500 m, and 100 m.

(iii) Estimation of E-value

In order to quantify the impact of unmeasured variables on our findings{Haneuse, 2019 #3865}, we have estimated the E-value for our primary analysis using the E-value calculator accessed at the link: https://www.evalue-calculator.com/evalue/.

© 2024 Bliuc D et al. *JAMA Network Open.*

eTable 1. Incidence of Fractures According to Age and Sex

	Women			Men		
Age, years	Fractures	Person-years	Rate/1000 (95%CI)	Fractures	Person-years	Rate/1000 (95%CI)
All	7190	603,005	11.92 (11.65 - 12.20)	4267	538,065	7.93 (7.70 - 8.18)
45-49	503	95,650	5.26 (4.82- 5.74)	403	63,187	6.38 (5.78 - 7.03)
50-59	1742	223,480	7.79 (7.44 - 8.17)	985	173,288	5.68 (5.34 - 6.05)
60-69	1720	162,646	10.58 (10.09 - 11.09)	973	158,294	6.15 (5.77 - 6.55)
70-79	1371	77,407	17.71 (16.80 - 18.67)	855	93,378	9.16 (8.56 - 9.79)
80+	1854	43,822	42.31 (40.43 - 44.28)	1051	49,918	21.05 (19.82 - 22.37)

© 2024 Bliuc D et al. *JAMA Network Open.*

eTable 2. Associations Between 1000 m Walking Limitation and Fracture Risk According to Sex and Age

	Women			Men		
**Outcome**	**Unadjusted**	**Model 1**	**Model 2**	**Unadjusted**	**Model 1**	**Model 2**
**Any fracture**						
**<70**						
Walking limitation						
Not at all	Reference	Reference	Reference	Reference	Reference	Reference
A little	1.42 (1.30 - 1.56)	1.28 (1.16 - 1.41)	1.19 (1.08 - 1.31)	1.37 (1.20 - 1.57)	1.27 (1.11 - 1.45)	1.16 (1.01 - 1.34)
A lot	1.94 (1.76 - 2.15)	1.64 (1.48 - 1.83)	1.39 (1.23 - 1.56)	2.25 (1.97 - 2.57)	1.89 (1.64 - 2.17)	1.65 (1.41 - 1.95)
**≥70**						
Walking limitation						
Not at all	Reference	Reference	Reference	Reference	Reference	Reference
A little	1.53 (1.40 - 1.68)	1.26 (1.17 - 1.36)	1.43 (1.30 - 1.57)	1.89 (1.69 - 2.12)	1.54 (1.37 - 1.73)	1.53 (1.34 - 1.72)
A lot	2.09 (1.93 - 2.26)	1.69 (1.57 - 1.83)	1.67 (1.53 - 1.84)	2.83 (2.55 - 3.14)	1.93 (2.18 - 2.71)	1.91 (1.69 - 2.16)

Numbers represent HR (95% CI); Model 1 is adjusted for variables included in the Garvan fracture risk calculator (weight, prior fracture and falls); Model 2 is adjusted for baseline comorbidities, marital status, education, smoking, and nursing home residency

© 2024 Bliuc D et al. *JAMA Network Open.*

eTable 3. Baseline Characteristics for Participants Stratified According to 1000, 500, and 100 m Walking Distance Limitation

Characteristics	Women					Men				
	No Limited mobility	Limit 1000 m	Limit 500m	Limited 100 m		No Limited mobility	Limited 1000 m	Limit 500m	Limit 100m	
	n (%)/mean (SD)	n (%)/mean (SD)	n (%)/mean (SD)	n (%)/mean (SD)	p-value	n (%)/mean (SD)	n (%)/mean (SD)	n (%)/mean (SD)	n (%)/mean (SD)	p-value
Number	95644	14494	8456	7150		89667	10614	6352	5803	
Age, years	59 (9)	65 (11)	68 (13)	70 (13)	<0.0001	62 (10)	68 (11)	70 (12)	70 (12)	<0.0001
Weight, kg	69 (14)	76 (20)	75 (19)	70 (13)	<0.0001	84 (15)	86 (18)	87 (19)	87 (20)	<0.0001
Falls	15578 (16)	1996 (28)	2716 (32)	5769 (40)	<0.0001	8884 (10)	1273 (22)	1755 (28)	3915 (37)	<0.0001
Prior fracture	10171 (11)	1124 (16)	1429 (17)	3090 (21)	<0.0001	6861 (8)	571 (10)	692 (11)	1400 (13)	<0.0001
Baseline comorbidities										
Heart failure	5029 (5)	957 (13)	1377 (16)	2949 (20)	<0.0001	11548 (13)	1544 (27)	1868 (29)	3397 (32)	<0.0001
Diabetes	4607 (5)	770 (11)	1172 (14)	2559 (18)	<0.0001	7665 (9)	963 (17)	1233 (19)	2414 (23)	<0.0001
Parkinson’s disease	265 (0.3)	53 (0.7)	95 (1)	221 (2)	<0.0001	394 (0.4)	80 (1)	95 (2)	273 (3)	<0.0001
Anxiety	8971 (11)	934 (15)	1228 (16)	2158 (17)	<0.0001	4930 (6)	506 (10)	595 (11)	1087 (12)	<0.0001
Asthma	9926 (12)	1163 (19)	1411 (19)	2699 (12)	<0.0001	6998 (9)	611 (12)	658 (12)	1217 (14)	<0.0001
Cancer	8195 (9)	865 (12)	967 (11)	1786 (12)	<0.0001	5712 (6)	916 (9)	541 (9)	540 (9)	<0.0001
Depression	13605 (17)	1441 (23)	1825 (25)	3309 (26)	<0.0001	7870 (10)	811 (16)	934 (17)	1857 (21)	<0.0001
Stroke	1304 (1)	247 (3)	504 (6)	1207 (8)	<0.0001	2011 (2)	364 (6)	526 (8)	1250 (12)	<0.0001
Private health insurance	66460 (69)	4174 (58)	4269 (50)	6382 (44)	<0.0001	62295 (69)	3190 (55)	2956 (47)	4275 (40)	<0.0001
Married/having a partner	71893 (75)	4657 (65)	4923 (58)	7562 (52)	<0.0001	74257 (84)	4508 (78)	4616 (73)	7488 (71)	<0.0001
Nursing home residency	1466 (2)	149 (2)	295 (3)	796 (5)	<0.0001	1674 (2)	134 (2)	194 (3)	505 (5)	<0.0001
Smoking	5992 (6)	578 (8)	724 (9)	1191 (8)	<0.0001	5945 (7)	562 (10)	680 (11)	1136 (11)	<0.0001

© 2024 Bliuc D et al. *JAMA Network Open.*

eTable 4. Associations Between 100 m, 500 m, and1000 m Walking Limitation and Fracture Risk According to Sex

	Women			Men		
**Outcome**	**Unadjusted**	**Model 1**	**Model 2**	**Unadjusted**	**Model 1**	**Model 2**
**Any fracture**						
Walking limitation						
None	Reference	Reference	Reference	Reference	Reference	Reference
100m	2.87 (2.71 - 3.04)	1.60 (1.50 - 1.71)	1.30 (1.20 - 1.41)	2.97 (2.74 - 3.21)	2.00 (1.84 - 2.18)	1.66 (1.49 - 1.85)
500m	2.26 (2.10 - 2.44)	1.43 (1.32 - 1.55)	1.26 (1.15 - 1.38)	2.22 (2.01 - 2.47)	1.62 (1.45 - 1.80)	1.40 (1.23 - 1.59)
1000m	1.65 (1.51 - 1.81)	1.20 (1.09 - 1.32)	1.10 (1.00 - 1.22)	1.70 (1.51 - 1.92)	1.32 (1.17 - 1.55)	1.20 (1.10 - 1.45)

Numbers represents hazard ratios (95% CI); Models 1 and 2 are adjusted for age; Model 1 is adjusted for variables included in the Garvan fracture risk calculator (age, weight, prior fracture, falls); Model 2 is adjusted for age, all comorbidities at baseline, number of medications, bisphosphonate use, marital status, education, and nursing home residency

eFigure 1. Forest Plot of Sex-Specific Hazard Ratios Comparing Fracture Risk for 1000 m Walking Limitation

Walking limitation	Hazard Ratio (95%CI)	P Value
Men		
A Lot	2.03 (1.86 - 2.22)	>.001
A Little	1.46 (1.34 - 1.60)	>.001
Women		
A Lot	1.60 (1.49 - 1.71)	>.001
A Little	1.32 (1.23 - 1.41)	>.001

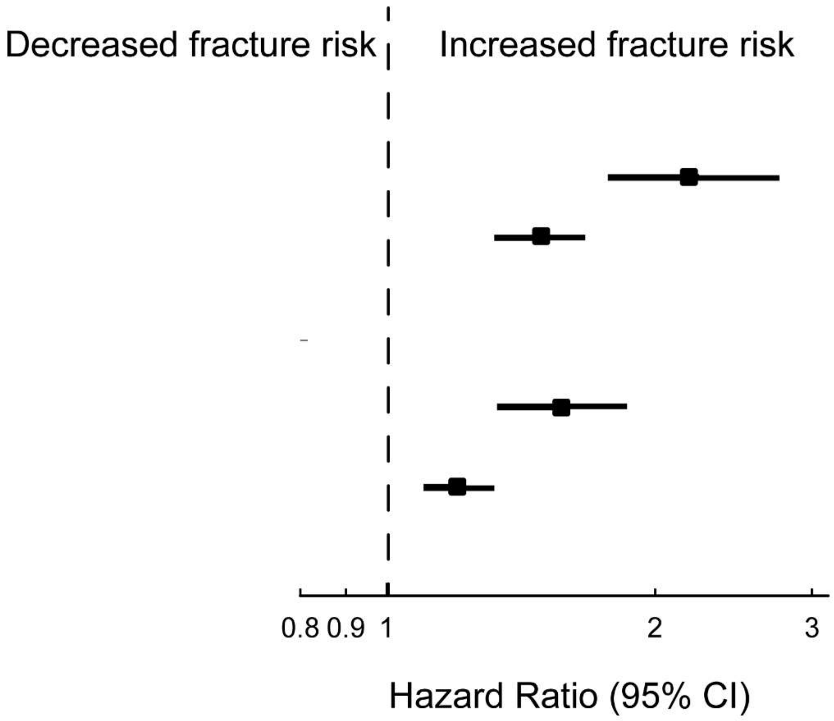

© 2024 Bliuc D et al. *JAMA Network Open.*

eFigure 2. Forest Plot of Sex-Specific Hazard Ratios Comparing Fracture Risk According to Walking Limitation at Different Distances of 1000 m, 500 m, and 100 m

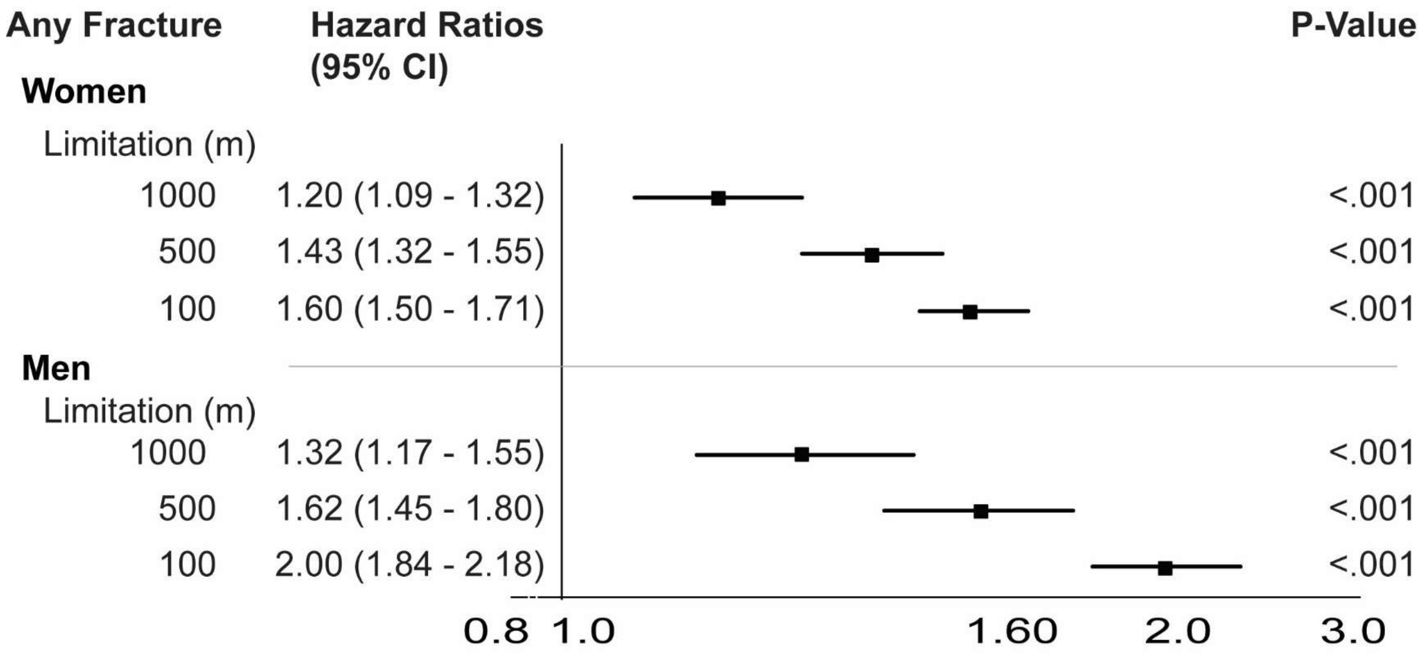

© 2024 Bliuc D et al. *JAMA Network Open.*
