# Supplementary material for: Patient Self-Assessment of Walking Ability and Fracture Risk in Older Australian Adults
Source: JAMA Netw Open. 2024 Jan 23;7(1):e2352675. doi: 10.1001/jamanetworkopen.2023.52675 (PMC10807297; doi:10.1001/jamanetworkopen.2023.52675)
Supplement: Supplement 2. — Data Sharing Statement [file jamanetwopen-e2352675-s002.pdf]

Data Sharing Statement

Bliuc. Patient Self-Assessment of Walking Ability and Fracture Risk in Older Australian Adults. *JAMA Netw Open*. Published January 23, 2024. doi:10.1001/jamanetworkopen.2023.52675

Data
Data available: No

Additional Information
Explanation for why data not available: Dana Bliuc and Jacqueline R Center had full access to all the data in the study and take responsibility for the integrity of the data and the accuracy of the data analysis.
